# Trajectories of loneliness, social isolation, and depressive symptoms before and after onset of pain in middle-aged and older adults

**DOI:** 10.1016/j.eclinm.2025.103209

**Published:** 2025-05-20

**Authors:** Mikaela Bloomberg, Feifei Bu, Daisy Fancourt, Andrew Steptoe

**Affiliations:** a Department of Epidemiology and Public Health, University College London, London, UK; b Department of Behavioural Science and Health, University College London, London, UK

**Keywords:** Pain, Chronic pain, Depression, Social isolation, Loneliness

## Abstract

**Background:**

Chronic pain is associated with poor psychosocial wellbeing; how loneliness, social isolation, and depressive symptoms evolve leading up to and following pain onset is unclear. We examined trajectories of these outcomes before and after pain onset.

**Methods:**

We analysed data from participants aged ≥50 years from the English Longitudinal Study of Ageing (ELSA). Data collection began in 2002 (wave 1) and was repeated biennially until 2019 (wave 9); wave 10 took place in 2021/23. Participants who had data from at least 2 waves were included. Participants reporting pain between 2002 and 2023 (‘pain group’) were matched with an equivalent number without pain (‘no-pain group’; N = 7336 total). Psychosocial outcomes were also assessed at each wave: Loneliness using the three-item subscale from the revised UCLA loneliness scale; social isolation using a score ranging from 0 to 5, with a higher score indicating more severe social isolation; and depression using the 8-item Center for Epidemiologic Studies Depression Scale. Piecewise linear mixed models were used to produce trajectories of loneliness, social isolation, and depressive symptom scores before and after pain onset and during the comparable time period in the no-pain group. Subgroup analyses examined how results differed by age, sex, education, and wealth.

**Findings:**

Loneliness and depressive symptoms were more severe for the pain group than the no-pain group before pain onset (e.g., difference [pain-no pain] eight years before pain onset = 0.19 points, 95% confidence interval = 0.11–0.28 for loneliness; difference = 0.14, 0.06–0.22 for depressive symptoms). For loneliness, this difference increased consistently during the study period (e.g., difference = 0.33, 0.26–0.40 eight years after pain onset). For depressive symptoms, the difference increased sharply to 0.58 (0.52–0.65) at pain onset and remained stable thereafter. Differences in depressive symptoms were most pronounced in less educated and less wealthy participants. There were negligible differences between pain and no-pain groups for social isolation.

**Interpretation:**

Loneliness and depressive symptoms progressively increased in severity years before pain onset. A holistic approach to pain is needed, incorporating early psychosocial interventions and targeted strategies for vulnerable populations.

**Funding:**

Nuffield Foundation Oliver Bird Fund, 10.13039/501100012041Versus Arthritis.

**Research in context**

**Evidence before this study**

We searched PubMed for publications until 7 January 2025 using search terms “chronic pain”, “depression”, “social isolation”, and “loneliness.” As a leading cause of disability, chronic pain is also associated with adverse social and mental health outcomes including loneliness, social isolation, and depression. Evidence suggests a bidirectional relationship of loneliness and depression with chronic pain, where individuals with chronic pain are more likely to be lonely and have depression and vice versa, whilst findings for social isolation are mixed.

**Added value of this study**

Understanding the complex interrelationships of chronic pain with social and psychological factors is necessary to design targeted pain management therapies. Neither cross-sectional nor longitudinal studies examining associations of loneliness, social isolation, and depression with chronic pain examine the evolution of these outcomes leading up to onset of pain, nor how they evolve following pain onset. This information is needed to understand optimal timing for integrating mental health and social support into the management of pain. In the present study, we examine how severity of loneliness, social isolation, and depressive symptoms progress leading up to and following onset of pain compared with a matched control group not experiencing pain over a follow-up period of up to 21 years, with consideration given to differences in results by age, sex, education level, and wealth.

**Implications of all the available evidence**

This study provides further evidence supporting the importance of targeting loneliness and mental health in older people. Proactive mental health and social support is needed in the decade preceding onset of pain and should be integrated into long-term pain management strategies, particularly for individuals with fewer socioeconomic resources.

## Introduction

Chronic pain is a major public health concern affecting an estimated 20–40% of adults in the UK and Europe and is among the leading causes of disability.^1^^,^^2^ Because chronic pain can profoundly impact mental health and wellbeing, it is best understood using a biopsychosocial model, which highlights the importance of considering psychosocial factors alongside biological factors in pain management.^3^ Loneliness, social isolation, and depression are key psychosocial factors that have been linked with chronic pain. Previous studies indicate that individuals with chronic pain are more likely to experience loneliness (a subjective feeling of lacking social connection^4^), though not necessarily greater social isolation (an objective lack of interaction with others^4^).^5^, ^6^, ^7^, ^8^, ^9^, ^10^, ^11^, ^12^, ^13^, ^14^ Emerging longitudinal evidence suggests the link between loneliness and chronic pain is bidirectional.^10^, ^11^, ^12^, ^13^, ^14^ Furthermore, loneliness and social isolation are associated with depression,^15^ which itself has a bidirectional relationship with chronic pain^16^ mediated by inflammatory and neuropathic mechanisms.^17^, ^18^, ^19^

Neither cross-sectional nor longitudinal studies examining loneliness, social isolation, depression, and chronic pain describe how psychosocial outcomes evolve in the period leading up to and following onset of chronic pain. A more nuanced understanding of the progression of these outcomes with consideration given to variation by demographic and socioeconomic characteristics is needed to design targeted prevention strategies and understand optimal timing for integrating mental health and social support into the management of chronic pain. This is particularly important for middle aged and older adults, as evidence suggests prevalence of chronic pain^1^ and depression^20^ increases with age and older adults may be particularly vulnerable to loneliness and social isolation.^21^

To address these gaps, we used data from 7336 participants aged ≥50 years from the English Longitudinal Study of Ageing (ELSA). We examined the longitudinal trajectories of loneliness, social isolation, and depressive symptoms leading up to and following onset of pain, compared with a matched control group of participants who did not report pain during a comparable time period. A secondary objective was to determine how these trajectories varied by demographic and socioeconomic characteristics.

## Methods

### Data sources

ELSA is a nationally-representative cohort study of adults aged ≥50 years living in private households in England. The first wave of ELSA was recruited from the Health Survey for England years 1998, 1999, and 2001, with household members aged 50 years and above eligible for inclusion. Data collection began in 2002/03 (wave 1) and was repeated biennially thereafter until 2018/19 (wave 9); wave 10 took place in 2021/23. Refreshment cohorts were recruited from subsequent years of the Health Survey for England at waves 3, 4, 6, 9, and 10. Further details of survey design and data collection are available elsewhere.^22^ Waves 1–10 (2002/03–2021/23) were included in the present study, with all ELSA respondents aged ≥50 years who did not report prevalent pain at their first interview and had at least two waves of data collection eligible for inclusion.

ELSA received ethical approval most recently from the South Central–Berkshire Research Ethics Committee on 22nd March 2021 (21/SC/0030). Written informed consent was granted at each interview. No further ethical approval was required for the present study.

### Pain assessment

At each wave, participants were asked to report whether they were “often troubled with pain” (yes or no) and the severity of pain (mild, moderate, or severe). Participants with moderate or severe pain during the follow-up period were categorised as the ‘pain’ group (also referred to as ‘cases’). Participants were asked to report where they felt their pain (all over, back, knee, hip, foot, mouth/tooth, or elsewhere). The ELSA survey does not regularly assess pain duration; as such, we could not confirm that reported pain met the general definition of chronicity (i.e., lasting for three months or longer^23^). Nevertheless, acute and sub-chronic pain can have a substantial impact on daily life and wellbeing.^24^

### Psychosocial outcomes

Psychosocial outcomes were assessed at each interview. Loneliness was assessed using the three-item subscale from the revised UCLA loneliness scale.^25^ Participants were asked to report frequency of feeling left out, isolated, or lacking companionship (“hardly or never”, “some of the time”, or “often”). Scores ranged from 3 to 9, with a higher score indicating more severe loneliness.

As in previous studies,^26^^,^^27^ a social isolation score was produced by giving one point for being unmarried and/or not cohabiting, up to three points for less than monthly contact (face-to-face, written, or telephone) with children, relatives, and/or friends, with one point given for each children, relatives, and friends, and one point for not being in any social organisations such as religious groups or clubs. Scores ranged from 0 to 5, with a higher score indicating more severe social isolation.

Finally, depressive symptoms were assessed using the 8-item Center for Epidemiologic Studies Depression (CES-D) Scale,^28^ which requires participants to report whether they often: 1) feel depressed; 2) feel everything is an effort; 3) have restless sleep; 4) are happy; 5) feel lonely; 6) feel sad; 7) cannot get going; and 8) enjoy life. One point was scored for each item with ‘happy’ and ‘enjoyed life’ reverse scored. A higher score indicated more depressive symptoms (range: 0–8).

### Covariates

Covariates were selected to ensure the pain group would be similar to the matched comparison group with respect to demographic and socioeconomic characteristics, health status, and health behaviours before onset of pain. Demographic and socioeconomic characteristics included age in years, birth year, sex (male or female; ELSA survey materials refer to sex not gender), baseline education level (less than high school diploma, high school diploma, or above high school diploma), and baseline wealth (net non-pension wealth, categorised into tertiles with the first tertile corresponding to the least wealth).

Other covariates assessed at baseline included self-report of clinical diagnosis (yes or no) of high blood pressure, diabetes, cancer, lung disease, heart disease, stroke, psychiatric conditions, osteoporosis, or arthritis; physical activity level (whether reports engaging in moderate-to-vigorous physical activity [MVPA] at least weekly or not); alcohol consumption (whether currently consumes any alcohol or not); and smoking (current smoker or not). Finally, we produced a wave 10 indicator, a dichotomous variable indicating whether a given interview took place at wave 10. This was done to account for the fact that wave 10 occurred during the COVID-19 pandemic when social contact patterns might have changed substantially from previous waves.

### Matching

To account for general changes in loneliness, social isolation, and depressive symptoms with age, we compared trajectories of psychosocial outcomes in the pain group with those in a control group drawn from ELSA participants who did not report pain during follow-up. The control group (the ‘no-pain’ group) was selected using coarsened exact matching^29^ based on age at baseline (five-year age groups), sex (male or female), birth year (five-year birth cohorts), and education level (less than high school diploma, high school diploma, or above high school diploma). Coarsened exact matching is robust to measurement error, requires fewer assumptions than other matching methods, and does not require the iterative checking process necessary to guarantee balance in other matching methods.^29^

Matching was implemented using the *cem* package^30^ in Stata with the *k*-to-*k* procedure used to produce an equal number of participants in the pain (cases) and no-pain (controls) groups, facilitating causal inference without the inclusion of model weights. In this procedure, controls were assigned to a matching stratum of cases with similar characteristics. Where the number of cases was fewer than the number of controls in a given matching stratum, controls were randomly dropped to give equal numbers of cases and controls. Because matching on additional variables leads to fewer matches and thus more participants being dropped from the analysis, which might reduce the generalisability of the results, matching criteria were limited to key demographic and socioeconomic characteristics. Other variables that might lead to differences between the pain and no-pain groups were accounted for by model adjustment.

### Statistical methods

We plotted raw outcome trajectories over time using local polynomial modelling with a fifth-degree polynomial to visually inspect trajectories in the pain and no-pain groups (Appendix A, Figures A2-A4). We then used piecewise linear mixed models to examine trajectories of loneliness, social isolation, and depressive symptoms before and after first pain onset. Mixed models use all available data regardless of length of follow-up, handle non-monotone missingness patterns, and item missingness assuming data missing-at-random.^31^ Though linear mixed models assume residuals are normally distributed which may not hold when modelling scores with a finite range as in the present study (Appendix A, Figure A1), when applied to large datasets, these models are nonetheless highly robust to violations to this assumption without substantively threatening inference.^32^ All models included a random intercept and slopes at the individual level (with an unstructured covariance matrix) to account for interindividual variation in trajectories.

In piecewise regression modelling, the slope of a line is allowed to differ before and after a pre-defined point (denoted time t=0), and each participant's follow-up period is centred at t=0. Time was assigned the value of 0 at the age when participants first reported pain for cases, or for controls, the median age at pain onset for cases in their matching stratum. Age at t=0 is denoted $age_{t=0}$.

Two time terms are included in each model to correspond to the periods before and after the split point at t=0, denoted *pre time* (t<0) and *post time* (t≥0). All models included group (pain or no pain), *pre time*, *post time*, and the interaction of group with *pre time* and *post time* to be able to examine the difference between groups in outcome trajectories before and after pain onset. Models were adjusted for the following covariates: $age_{t=0}$, sex, birth year, education, wealth, chronic conditions, physical activity, alcohol consumption, smoking status, and the wave 10 indicator. All covariates except $age_{t=0}$ and birth year were treated as categorical variables. We also examined whether to include nonlinear *pre time* and *post time* (quadratic or cubic), and interactions of nonlinear terms with group (pain or no pain); we retained nonlinear terms if significant (p < 0.05) on the basis of the Wald test. We included $pre\;time^{2}$ and $post\;time^{2}$ in the models for depressive symptoms; all other models had linear *pre time* and *post time* terms only.

We examined whether differences between the pain and no-pain groups in outcome trajectories varied by $age_{t=0}$ (<65 or 65+ years), sex, education level, or wealth tertile by additionally including in models interactions of each of these characteristics with group (pain or no-pain), *pre time*, and *post time*. Separate models were fitted for each of these demographic and socioeconomic characteristics. Wealth and education were treated as ordinal variables in these analyses to increase statistical power.

We used these models to produce eight-year trajectories for each outcome (loneliness score, social isolation score, and depressive symptom score) in the pain and no-pain groups before and from t=0, corresponding to 16 years of follow-up total which was the 75th percentile for follow-up duration. We reported the difference between groups in each score at t=−8, t=0, and t=8, and differences in trajectory before and after pain onset. Finally, we reported whether differences between pain and no-pain groups for each psychosocial outcome varied by $age_{t=0}$, sex, education, and wealth. All analyses were performed in StataMP 18.0, with a two-sided p < 0.05 considered significant.

### Additional analyses

Taking into consideration the difficulty of interpreting a one-point increase in loneliness, social isolation, or depressive symptom score, we also used piecewise logistic mixed models to examine prevalence of loneliness, social isolation, and depression in the pain and no-pain groups in order to further contextualise the main analyses. We dichotomised outcomes using previously established cut-offs relevant for health and wellbeing (loneliness score ≥6 indicating high loneliness,^33^ social isolation score ≥2 indicating social isolation,^34^ and CES-D score ≥3 indicating depression^28^). These models included all the same covariates as the main analysis and had a random intercept at the individual level. Unlike linear mixed models where population-average and individual-specific estimates are equivalent, odds ratios produced by logistic mixed models are individual-specific only. We used the transformation for random-intercept-only models described by Hedeker et al.^35^ (Appendix A, Methods A1) to produce population-average odds ratios for each outcome with the no-pain group as the reference at t=−8, t=0, and t=8 to complement the continuous results.

Finally, we conducted two sensitivity analyses: first, limiting the pain group to participants reporting pain across multiple waves (consecutive or non-consecutive) to assess longer-term pain that was more likely to meet the definition for chronicity; and second, expanding the pain group to include those with mild as well as moderate and severe pain.

### Role of the funding source

The funders were not involved in study design, the collection, analysis or interpretation of data, writing of the report, or the decision to submit the paper for publication.

## Results

### Participant characteristics

Of 21,221 ELSA participants aged ≥50 years, 644 (3.0%) were missing pain data, and 5487 (25.9%) had prevalent pain at baseline. Of the remaining 15,090, 3071 (20.4%) were missing loneliness, social isolation, or depressive symptom data for all waves of follow-up, 699 (4.6%) were missing other covariates, and 1649 (10.9%) had <2 waves of data; of the 9671 remaining (3921 [40.5%] reporting pain), 3668 participants with pain were matched with an equivalent number of controls, leading to 7336 participants being included in the analyses (Appendix A, Figure A5). Excluded ELSA respondents were generally similar with respect to demographic and socioeconomic characteristics to those included in the analysis, though they were wealthier and healthier (Appendix A, Table A1).

At baseline, participants in the pain and no-pain groups were similar with respect to age (mean 60.9 years for both; SD = 8.8 for no pain, SD = 8.9 for pain), birth year (median 1944 for both; IQR = 1936–51 for no pain, 1935–51 for pain), sex (54.6% female), education level (63.6% educated to high school level or above), alcohol consumption, smoking status, and prevalence of heart disease, stroke, and osteoporosis (Table 1). The pain group was less wealthy than the no-pain group (p < 0.0001), less likely to report weekly MVPA (p < 0.0001), and more likely to report high blood pressure (p < 0.0001), diabetes (p = 0.00086), cancer (p = 0.0094), lung disease (p = 0.0037), psychiatric conditions (p = 0.00035), and arthritis (p < 0.0001).

**Table 1:** Participant characteristics at baseline (N = 7336).

	No pain N = 3668	Pain N = 3668	p-value
Age, mean (SD)	60.9 (8.8)	60.9 (8.9)	0.92
Birth year, median (IQR)	1944 (1936–51)	1944 (1935–51)	0.89
Sex			
Male	1664 (45.4)	1664 (45.4)	>0.99
Female	2004 (54.6)	2004 (54.6)	
Education level			
Low	1334 (36.4)	1334 (36.4)	
Intermediate	1782 (48.6)	1782 (48.6)	>0.99
High	552 (15.0)	552 (15.0)	
Wealth tertile			
Low wealth	1220 (33.3)	1485 (40.5)	
Intermediate wealth	1245 (33.9)	1147 (31.3)	<0.0001
High wealth	1203 (32.8)	1036 (28.2)	
Reports weekly MVPA	3205 (87.4)	3076 (83.9)	<0.0001
Consumes alcohol	3405 (92.8)	3376 (92.0)	0.22
Smokes	592 (16.1)	646 (17.6)	0.099
Reports diagnosis of			
High blood pressure	1013 (27.6)	1177 (32.1)	<0.0001
Diabetes	145 (4.0)	207 (5.6)	0.00086
Cancer	148 (4.0)	196 (5.3)	0.0094
Lung disease	86 (2.3)	129 (3.5)	0.0037
Heart disease	373 (10.2)	422 (11.5)	0.071
Stroke	59 (1.6)	75 (2.0)	0.19
Psychiatric conditions	191 (5.2)	266 (7.3)	0.00035
Osteoporosis	69 (1.9)	84 (2.3)	0.35
Arthritis	402 (11.0)	929 (25.3)	<0.0001
High loneliness	172 (15.0)	251 (22.2)	<0.0001
Social isolation	1284 (43.1)	1289 (43.7)	0.64
Depression	408 (11.1)	719 (19.6)	<0.0001
Loneliness score	4.0 (1.3)	4.3 (1.6)	<0.0001
Social isolation score	1.4 (1.0)	1.4 (1.0)	0.69
Depressive symptom score	0.90 (1.5)	1.4 (1.8)	<0.0001

Data shown are N (%) unless otherwise indicated. Low education = less than high school; intermediate = high school diploma, high = above high school diploma.

Abbreviations: SD, standard deviation; IQR, interquartile range; MVPA, moderate-to-vigorous physical activity.

The distributions of psychosocial scores at baseline are shown in the Supplementary materials (Appendix A, Figure A6). At baseline, the pain group was more likely than the no-pain group to be lonely (p < 0.0001) and have CES-D scores consistent with depression (p < 0.0001), but was similar to the no-pain group with respect to social isolation. Follow-up duration before and after onset of pain or during the equivalent period was similar for the pain (median before = 5.5 years, interquartile range [IQR] = 4.0–6.5; median after = 6.0 years, IQR = 2.0–11.0) and no-pain (median before = 5.0 years, IQR = 3.0–9.0; median after = 5.0 years, IQR = 0.0–10.0) groups. The mean age at t=0 was similar between groups (66.4 years, SD = 8.5 for no pain and 67.3 years, SD = 9.5 for the pain group). Of 3533 participants in the pain group, at first report of pain, 66 (1.9%) reported pain all over, 2715 (76.9%) reported back, knee, hip, or foot pain, 19 (0.5%) reported mouth/tooth pain only, and 733 (20.7%) reported pain elsewhere only.

### Loneliness

**Fig. 1:**
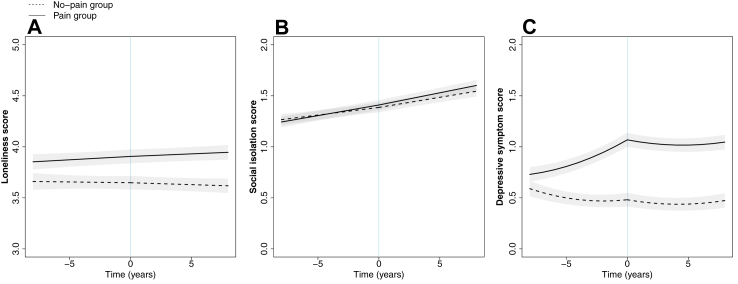
**Trajectories of loneliness, social isolation, and depressive symptom score before and after onset of pain**. Panel A shows loneliness score (range: 3–9), panel B shows social isolation score (range: 0–5), and panel C shows depressive symptom score (range: 0–8). Models are adjusted for $age_{t=0}$, sex, birth year, education, wealth, chronic conditions, physical activity, alcohol consumption, smoking status, and wave 10 indicator. Plotted for reference values of covariates ($age_{t=0}$ = 65, male, born 1940–49, high school diploma, mean wealth, no chronic conditions, weekly moderate-vigorous physical activity, consumes alcohol, non-smoker, not wave 10).

After adjustment for covariates, severity of loneliness differed between the pain and no-pain groups for the entire study period (Fig. 1, panel A). Eight years before pain onset, the pain group had loneliness scores 0.19 points (0.11–0.28) higher than the no-pain group (p < 0.0001) and had 1.29 times the odds of experiencing high loneliness compared with the no-pain group (95% confidence interval [CI] = 1.09–1.53; p = 0.0035).

Severity of loneliness increased in the period leading up to the onset of pain, a trend not seen in the no-pain group, though this difference in trajectories between the pain and no-pain groups did not reach statistical significance in the period before onset of pain (p = 0.17). The difference in loneliness between the pain and no-pain groups was 0.26 points (0.19–0.32; p < 0.0001) at the time of pain onset. The pain group had 1.68 (1.51–1.87) times the odds of experiencing high loneliness at pain onset compared with the no-pain group (p < 0.0001).

Following onset of pain, the difference in loneliness trajectories between the pain and no-pain group reached statistical significance (p = 0.032), where the severity of loneliness continued to increase modestly in the pain group, but not in the no-pain group. The difference in loneliness score increased to 0.33 points (0.26–0.40) eight years after pain onset (p < 0.0001). At this time, participants in the pain group had 1.61 (1.45–1.79) times the odds of experiencing high loneliness compared with the no-pain group (p < 0.0001).

### Social isolation

After adjustment for covariates, there were negligible differences in severity of social isolation (Fig. 1, panel B) between the pain and no-pain groups either before pain onset (difference [pain-no pain] eight years before onset = −0.02, −0.08–0.03; p = 0.40) or at pain onset (difference = 0.02, −0.02–0.07; p = 0.33); there was a minor difference eight years after pain onset (difference = 0.06, 0.00–0.11; p = 0.040). Degree of social isolation increased over time for both the pain and no-pain groups. The corresponding odds ratios for experiencing social isolation in the pain group compared with the no-pain group were 0.94 (0.84–1.06; p = 0.33) eight years before pain onset, 1.04 (0.95–1.14; p = 0.43) at pain onset, and 1.01 (0.91–1.11; p = 0.91) eight years after pain onset.

### Depressive symptoms

After adjustment for covariates, severity of depressive symptoms differed between the pain and no-pain groups for the entire study period (Fig. 1, panel C). Eight years before first onset of pain, the pain group had depressive symptom scores 0.14 points (0.06–0.22) higher than the no-pain group (p = 0.00078) and had 1.29 (1.10–1.51) times the odds of depression compared with the no-pain group (p = 0.0016).

The depressive symptom score trajectory leading up to pain onset or during the comparable time period in the no-pain group differed between the pain and no-pain groups (p < 0.0001), where the depressive symptom score increased rapidly for the pain group but not for the no-pain group. As a result, the difference in depressive symptom score at pain onset more than tripled to 0.58 points (0.52–0.65; p < 0.0001). At onset of pain, the pain group had 2.33 (2.09–2.59) times the odds of depression compared with the no-pain group (p < 0.0001).

Following pain onset, depressive symptom score trajectories were similar between groups (p = 0.71) and remained relatively stable. Eight years after onset of pain, the difference between groups was maintained at 0.57 points (0.49–0.64; p < 0.0001). At this time, participants in the pain group still had 2.21 (1.97–2.47) times the odds of depression compared with the no-pain group (p < 0.0001).

### Demographic and socioeconomic differences

**Fig. 2:**
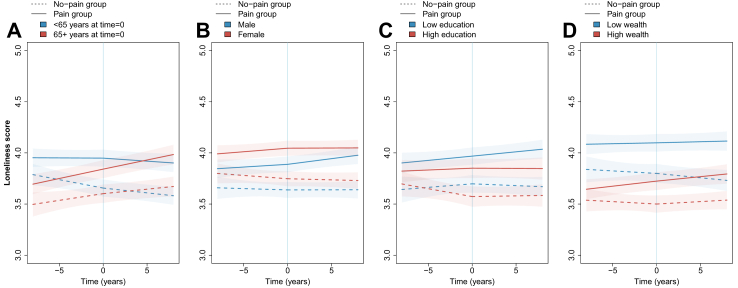
**Loneliness score trajectory before and after onset of pain for different demographic and socioeconomic characteristics**. Plotted based on models including $age_{t=0}$, sex, birth year, education, wealth, chronic conditions, physical activity, alcohol consumption, smoking status, and wave 10 indicator. Panel A model also interactions between group (pain or no pain) and age at t=0 (<65 or 65+). Panel B model includes interactions between group and sex (male or female). Panel C model includes interactions between group and education (less than high school, high school diploma, or above high school diploma, with results plotted for less than high school [low] and above high school [high]). Panel D includes interactions between group and wealth (in tertiles, plotted for lowest wealth [low] and highest wealth [high] tertile).

**Fig. 3:**
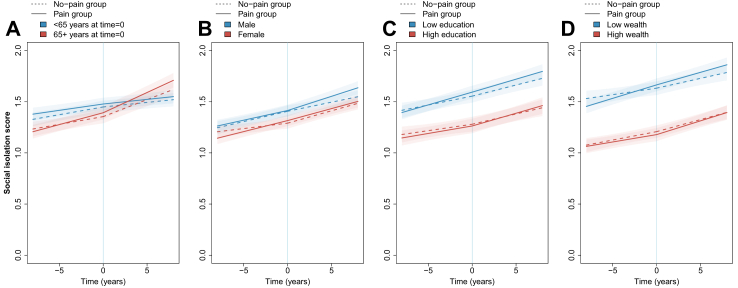
**Social isolation score trajectory before and after onset of pain plotted for different demographic and socioeconomic characteristics**. Plotted based on models including $age_{t=0}$, sex, birth year, education, wealth, chronic conditions, physical activity, alcohol consumption, smoking status, and wave 10 indicator. Panel A model also interactions between group (pain or no pain) and age group at t=0 (<65 or 65+). Panel B model includes interactions between group and sex (male or female). Panel C model includes interactions between group and education (less than high school, high school diploma, or above high school diploma, with results plotted for less than high school [low] and above high school [high]). Panel D includes interactions between group and wealth (in tertiles, plotted for lowest wealth [low] and highest wealth [high] tertile).

**Fig. 4:**
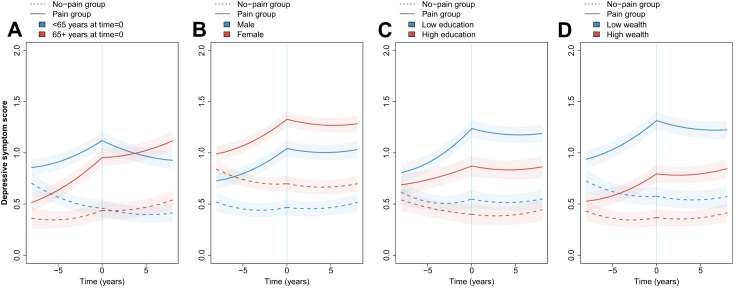
**Depressive symptom score trajectory before and after onset of pain plotted for different demographic and socioeconomic characteristics**. Plotted based on models including $age_{t=0}$, sex, birth year, education, wealth, chronic conditions, physical activity, alcohol consumption, smoking status, and wave 10 indicator. Panel A model also interactions between group (pain or no pain) and age group at t=0 (<65 or 65+). Panel B model includes interactions between group and sex (male or female). Panel C model includes interactions between group and education (less than high school, high school diploma, or above high school diploma, with results plotted for less than high school [low] and above high school [high]). Panel D includes interactions between group and wealth (in tertiles, plotted for lowest wealth [low] and highest wealth [high] tertile).

There was weak evidence that the difference between the pain and no-pain groups in loneliness or social isolation scores varied by age, sex, education level, or wealth (Fig. 2, Fig. 3; Appendix A, Table A2). However, the difference in depressive symptom scores between groups varied modestly by age and more substantially by education level and wealth (Fig. 4, Appendix A, Table A2). For example, at onset of pain, in all age, education, and wealth categories, the pain group had more depressive symptoms than the no-pain group. However, the difference between the pain and no-pain groups was 0.15 points (0.02–0.28) larger in the younger age category (<65 years) than in the older age category (p = 0.028), 0.32 points (0.04–0.61) larger in the low compared with the high education category (p = 0.026), and 0.47 points (0.23–0.70) larger in the lowest wealth tertile compared with the highest (p = 0.00010).

### Sensitivity analyses

Conclusions were substantially unchanged when analyses were restricted to participants reporting longer-term pain and when participants reporting mild pain were included in the pain group (Appendix A, Tables A3–A4, Figures A7–A8).

## Discussion

This examination of trajectories of loneliness, social isolation, and depressive symptoms in 7336 adults aged ≥50 with and without pain has four key results. First, the severity of depressive symptoms showed a sharply rising trend even eight years before onset of pain that was absent in the no-pain group during the same time period, then remained stable after pain onset. Second, severity of loneliness modestly increased in the pain group but not in the no-pain group across the entire study period. Third, there was little difference in social isolation between the pain and no-pain groups. Finally, the differences between the pain and no-pain groups in depressive symptoms were most pronounced in those with less education and less wealth. The results suggest a complex relationship of psychosocial wellbeing with pain that differs by socioeconomic position.

Psychosocial factors might contribute to pain by inducing psychological stress leading to greater susceptibility to inflammation, by dysregulating the autonomic nervous system causing increased sensitisation to peripheral signals such as pain, and by inducing maladaptive changes in immunological responses.^36^, ^37^, ^38^ Pain might then lead individuals to further withdraw and engage in fewer healthy behaviours, exacerbating poor psychosocial wellbeing. The present findings are consistent with evidence that suggests a bidirectional association of loneliness and depression with chronic pain,^10^, ^11^, ^12^, ^13^, ^14^^,^^39^, ^40^, ^41^, ^42^, ^43^ but add nuance to these previous studies by demonstrating the timing and severity of loneliness and depressive symptoms in the years before and after pain onset. We also highlight less educated and wealthy individuals as most vulnerable to poor mental wellbeing, likely in part because these individuals have fewer resources to support mental health and pain management.^44^

Though our findings for social isolation were in accordance with one previous study showing no association between social isolation and chronic pain,^8^ another cross-sectional study using wave 2 of ELSA found individuals experiencing mild, moderate, or severe pain were somewhat less likely to be socially isolated.^9^ These results are consistent with our sensitivity analysis including mild pain, though the difference between the pain and no-pain groups was still minor in this sensitivity analysis. Adjustment for variables along the causal pathway—such as depression—in the previous study might further contribute to differences in results. More research is needed to clarify how severity of pain affects relationships between psychosocial outcomes and pain.

The main strengths of this paper are that it makes use of longitudinal data from a nationally-representative cohort study with a long follow-up period and uses a matched control group to take into account ageing-related trends in the outcomes in the study population. Previous studies are cross-sectional or examine bidirectional associations between pain, loneliness, and depression with outcomes assessed at a single time point. With repeated measures collected over up to 21 years of follow-up, we were able to produce longitudinal trajectories before and after onset of pain. We also examined outcomes both as a score and dichotomously using previously defined cut-offs, expanding on previous studies that primarily use dichotomous outcomes.

There are also several limitations. The inclusion of a matched control group does not rule out the potential for unmeasured confounding threatening causality, though we accounted for key confounders by model adjustment to account for differences between cases and controls after matching. Though data were drawn from a nationally representative study, ELSA respondents were excluded during the matching process and due to missing data and findings are not themselves nationally representative. This does not necessarily bias the results, but may reduce the generalisability of the findings. In particular, participants included in the analysis are somewhat healthier than those excluded. As such, examining the generalisability of findings in other cohorts is a key area of future research. Some participants with incomplete follow-up who did not report pain during that period may have developed pain after being lost to follow-up. In this case, their observed trajectories would represent the period leading up to pain onset, despite their classification in the no-pain group. However, this would lead to an underestimation of differences in psychosocial outcome trajectories between the pain and no-pain groups and would not contradict the overall findings of the paper. Differential attrition could have impacted the results; however, follow-up duration is similar between pain groups suggesting differential attrition is less likely to have influenced the findings. Because of the way pain is assessed in ELSA, we could not distinguish chronic pain from other types of pain; nonetheless, results were consistent when we restricted analyses to individuals reporting two or more waves of pain, suggesting the conclusions are relevant for longer-term pain. Notably, depressive symptoms increased before pain onset and remained elevated afterward—a trend not seen in those not experiencing pain—indicating that even if many ELSA respondents experienced acute or sub-chronic pain, psychological effects persisted. We rely on self-reported chronic conditions at baseline, which may lead to some misclassification, though evidence suggests good concordance between self-report and medical records for these conditions.^45^ We were unable to include data on medication, surgery, or other treatments or therapies for pain during the follow-up period as these data were not available. Finally, participants in ELSA are 95% white, reflecting the demographic characteristics of over 50s in England during the ELSA study period (e.g., 94% white in 2011^46^). Future studies should examine outcomes in younger adults, with better racial and ethnic minority representation, should explore how specific types of pain differentially affect psychosocial wellbeing, and interactions between psychosocial outcomes.

Understanding the progression of psychosocial outcomes is necessary to design tailored therapies to improve quality of life for individuals experiencing pain. These findings highlight the need for early psychological intervention, as the rapid increase in depressive symptoms years before pain onset suggests an opportunity to mitigate or delay pain through targeted mental health programmes. The association of loneliness—but not social isolation—with pain suggests that interventions aimed at individuals with pain might prioritise enhancing perceived social support and connection, rather than simply increasing social interaction. Finally, the results point to the need to prioritise vulnerable populations with fewer socioeconomic resources through accessible mental health care and community support programmes.

The present study underscores the bidirectional nature of relationships between pain and mental health, highlighting progressive increases in loneliness and depressive symptoms well before onset of pain. Taken together, these findings point to the importance of a holistic approach to pain management that includes early psychological interventions, ongoing support systems, and targeted strategies for vulnerable populations.

## Contributors

Conceptualisation: DF, FB, AS.

Methodology: MB, FB, AS.

Validation: AS.

Formal analysis: MB.

Data curation: MB, AS.

Writing –original draft preparation: MB.

Writing –review and editing: All authors.

Visualisation: MB.

Supervision: AS.

Funding acquisition: DF, AS.

MB and AS accessed and verified the data. All authors had full access to the data and accept responsibility to submit for publication.

## Data sharing statement

ELSA data are available to researchers after registration with the UK data service at https://beta.ukdataservice.ac.uk/datacatalogue/series/series?id=200011.

## Declaration of interests

The authors have declared that no competing interests exist.
