# Supplementary material for: Trajectories of loneliness, social isolation, and depressive symptoms before and after onset of pain in middle-aged and older adults
Source: eClinicalMedicine. 2025 May 20;84:103209. doi: 10.1016/j.eclinm.2025.103209 (PMC12273840; doi:10.1016/j.eclinm.2025.103209)
Supplement: Supplementary Tables and Figures [file mmc1.pdf]

**<u>Appendix A: Supplementary materials</u>**

# Trajectories of loneliness, social isolation, and depressive symptoms before and after onset of chronic pain in middle-aged and older adults

Mikaela Bloomberg, PhD[1]
Feifei Bu, PhD[2]
Professor Daisy Fancourt, PhD[2]
Professor Andrew Steptoe, DSc[2]

[1]Department of Epidemiology and Public Health, University College London, London, UK
[2]Department of Behavioural Science and Health, University College London, London, UK

**Contents**

Methods A1. Derivation of population-average odds ratio. ................................................3

Table A1. Baseline characteristics of ELSA participants included vs. excluded from analysis. ........................................................................................................................4

Table A2. Variation in pain differences in loneliness, social isolation, and depressive symptoms by demographic and socioeconomic characteristics (N=7,336). .........................5

Table A3. Difference in loneliness, social isolation, and depressive symptom scores between pain groups when analyses are restricted to individuals with longer-term chronic pain (N=5,438). ..........................................................................................................6

Table A4. Difference in loneliness, social isolation, and depressive symptom scores between pain groups when individuals reporting mild pain are included in the pain group (N=6,696). ..................................................................................................................7

Figure A1. Q-Q plots for model residuals compared with normal distribution....................8

Figure A2. Loneliness score trajectory fitted using local polynomial regression. .................9

Figure A3. Social isolation score trajectory fitted using local polynomial regression. ........10

Figure A4. Depressive symptom score trajectory fitted using local polynomial regression. ......................................................................................................................11

Figure A5. Flowchart of sample selection..........................................................................12

Figure A6. Distribution of psychosocial scores at baseline.................................................13

Figure A7. Trajectories of loneliness, social isolation, and depressive symptom scores before and after onset of pain (longer-term chronic pain only). .......................................14

Figure A8. Trajectories of loneliness, social isolation, and depressive symptom scores before and after onset of pain (including mild pain).........................................................15

References .........................................................................................................................16

Methods A1. Derivation of population-average odds ratio.

Given an estimated logistic mixed model with a random intercept of variance $\widehat{\sigma}_v^2$ from which individual-specific coefficients, $\hat{\beta}^{ss}$, have been produced, we can estimate population-average coefficients, $\hat{\beta}^{pa}$, using the formula:[1]

$$\hat{\beta}^{pa} = \frac{\hat{\beta}^{ss}}{\sqrt{\frac{\widehat{\sigma}_v^2 + \frac{\pi^2}{3}}{\frac{\pi^2}{3}}}}$$

Table A1. Baseline characteristics of ELSA participants included vs. excluded from analysis.

| | **Excluded** N=13,885 | **Included** N=7,336 |
|---|---|---|
| Age, mean (SD) | 61.8 (10.2) | 60.9 (8.8) |
| Birth year, median (IQR) | 1944.0 (14.1) | 1942.9 (10.5) |
| Sex | | |
| Male | 5571 (40.1) | 3328 (45.4) |
| Female | 6416 (46.2) | 4008 (54.6) |
| Missing | 1898 (13.7) | 0 (0.0) |
| Education level | | |
| Low | 5062 (36.5) | 2668 (36.4) |
| Intermediate | 4884 (35.2) | 3564 (48.6) |
| High | 1977 (14.2) | 1104 (15.0) |
| Missing | 1962 (14.1) | 0 (0.0) |
| Wealth tertile | | |
| Low wealth | 5135 (37.0) | 2705 (36.9) |
| Intermediate wealth | 3509 (25.3) | 2392 (32.6) |
| High wealth | 2866 (20.6) | 2239 (30.5) |
| Missing | 2375 (17.1) | 0 (0.0) |
| Reports weekly MVPA | 8478 (61.1) | 6281 (85.6) |
| Consumes alcohol | 8651 (62.3) | 6781 (92.4) |
| Smokes | 2156 (15.5) | 1238 (16.9) |
| Reports diagnosis of: | | |
| High blood pressure | 3892 (28.0) | 2190 (29.9) |
| Diabetes | 980 (7.1) | 352 (4.8) |
| Cancer | 728 (5.2) | 344 (4.7) |
| Lung disease | 665 (4.8) | 215 (2.9) |
| Heart disease | 1918 (13.8) | 795 (10.8) |
| Stroke | 462 (3.3) | 134 (1.8) |
| Psychiatric conditions | 943 (6.8) | 457 (6.2) |
| Osteoporosis | 532 (3.8) | 153 (2.1) |
| Arthritis | 3701 (26.7) | 1331 (18.1) |

Data shown are N (%) unless otherwise indicated. Low education = less than high school; intermediate = high school diploma, high = above high school diploma.
Abbreviations: SD, standard deviation; IQR, interquartile range; MVPA, moderate-to-vigorous physical activity.

Table A2. Variation in pain differences in loneliness, social isolation, and depressive symptoms by demographic and socioeconomic characteristics (N=7,336).

| Outcome | Interaction between pain and: | $\beta$ (95% CI) | P-value |
|---|---|---|---|
| *Loneliness* | Age 65+ (ref: <65) | 0.01 (-0.08 to 0.10) | 0.85 |
| | Age 65+, $pre\ time$ | 0.01 (-0.00 to 0.02) | 0.12 |
| | Age 65+, $post\ time$ | 0.01 (-0.01 to 0.02) | 0.33 |
| | Female (ref: male) | 0.02 (-0.08 to 0.11) | 0.71 |
| | Female, $pre\ time$ | 0.01 (-0.00 to 0.03) | 0.078 |
| | Female, $post\ time$ | -0.01 (-0.02 to 0.00) | 0.10 |
| | High education (ref: low) | -0.08 (-0.29 to 0.12) | 0.42 |
| | High education, $pre\ time$ | -0.01 (-0.04 to 0.02) | 0.58 |
| | High education, $post\ time$ | 0.00 (-0.03 to 0.03) | 0.91 |
| | High wealth (ref: low) | -0.09 (-0.26 to 0.08) | 0.29 |
| | High wealth, $pre\ time$ | -0.02 (-0.05 to 0.00) | 0.067 |
| | High wealth, $post\ time$ | -0.00 (-0.03 to 0.02) | 0.80 |
| *Social isolation* | Age 65+ (ref: <65) | -0.05 (-0.18 to 0.07) | 0.42 |
| | Age 65+, $pre\ time$ | -0.01 (-0.03 to 0.01) | 0.38 |
| | Age 65+, $post\ time$ | 0.01 (-0.01 to 0.02) | 0.50 |
| | Female (ref: male) | 0.05 (-0.08 to 0.18) | 0.46 |
| | Female, $pre\ time$ | 0.01 (-0.02 to 0.03) | 0.65 |
| | Female, $post\ time$ | -0.01 (-0.02 to 0.01) | 0.31 |
| | High education (ref: low) | 0.01 (-0.27 to 0.29) | 0.94 |
| | High education, $pre\ time$ | 0.03 (-0.02 to 0.08) | 0.30 |
| | High education, $post\ time$ | -0.02 (-0.06 to 0.02) | 0.26 |
| | High wealth (ref: low) | -0.11 (-0.34 to 0.12) | 0.34 |
| | High wealth, $pre\ time$ | 0.01 (-0.03 to 0.06) | 0.59 |
| | High wealth, $post\ time$ | -0.01 (-0.04 to 0.02) | 0.51 |
| *Depressive symptoms* | Age 65+ (ref: <65) | -0.15 (-0.28 to -0.02) | 0.028 |
| | Age 65+, $pre\ time$ | -0.02 (-0.04 to 0.00) | 0.10 |
| | Age 65+, $post\ time$ | 0.03 (0.01 to 0.04) | 0.0044 |
| | Female (ref: male) | 0.05 (-0.08 to 0.18) | 0.41 |
| | Female, $pre\ time$ | 0.01 (-0.01 to 0.04) | 0.21 |
| | Female, $post\ time$ | 0.00 (-0.02 to 0.02) | 0.85 |
| | High education (ref: low) | -0.32 (-0.61 to -0.04) | 0.026 |
| | High education, $pre\ time$ | -0.03 (-0.08 to 0.02) | 0.21 |
| | High education, $post\ time$ | -0.00 (-0.04 to 0.04) | 0.96 |
| | High wealth (ref: low) | -0.47 (-0.70 to -0.23) | 0.00010 |
| | High wealth, $pre\ time$ | -0.04 (-0.08 to 0.00) | 0.077 |
| | High wealth, $post\ time$ | 0.02 (-0.02 to 0.05) | 0.33 |

Models include $age_{t=0}$, sex, birth year, education, wealth, chronic conditions, physical activity, alcohol consumption, smoking status, wave 10 indicator and all interaction terms listed in table, with separate models fitted for each demographic or socioeconomic characteristic of interest.

Table A3. Difference in loneliness, social isolation, and depressive symptom scores between pain groups when analyses are restricted to individuals with longer-term chronic pain (N=5,438).

| **Years from onset of pain** | **Difference, pain – no pain** (95% CI) | **P-value** |
|---|---|---|
| *Loneliness* | | |
| -8 | 0.23 (0.12 to 0.33) | <0.0001 |
| 0 | 0.29 (0.22 to 0.38) | <0.0001 |
| 8 | 0.38 (0.29 to 0.46) | <0.0001 |
| *Social isolation* | | |
| -8 | -0.03 (-0.09 to 0.04) | 0.39 |
| 0 | 0.01 (-0.05 to 0.07) | 0.72 |
| 8 | 0.00 (-0.06 to 0.07) | 0.89 |
| *Depressive symptoms* | | |
| -8 | 0.17 (0.07 to 0.27) | 0.00064 |
| 0 | 0.73 (0.65 to 0.81) | <0.0001 |
| 8 | 0.75 (0.66 to 0.83) | <0.0001 |

Pain group restricted to subsample who report pain at multiple waves following first report of pain (N=2,719 each cases and controls). Models include $age_{t=0}$, sex, birth year, education, wealth, chronic conditions, physical activity, alcohol consumption, smoking status, and wave 10 indicator.

Table A4. Difference in loneliness, social isolation, and depressive symptom scores between pain groups when individuals reporting mild pain are included in the pain group (N=6,696).

| **Years from onset of pain** | **Difference, pain – no pain** (95% CI) | **P-value** |
|---|---|---|
| *Loneliness* | | |
| -8 | 0.13 (0.04 to 0.22) | 0.0060 |
| 0 | 0.20 (0.13 to 0.26) | <0.0001 |
| 8 | 0.23 (0.16 to 0.31) | <0.0001 |
| *Social isolation* | | |
| -8 | -0.10 (-0.15 to 0.04) | 0.0013 |
| 0 | -0.02 (-0.07 to 0.03) | 0.43 |
| 8 | -0.01 (-0.07 to 0.05) | 0.71 |
| *Depressive symptoms* | | |
| -8 | 0.14 (0.06 to 0.23) | 0.0011 |
| 0 | 0.47 (0.41 to 0.54) | <0.0001 |
| 8 | 0.46 (0.39 to 0.53) | <0.0001 |

Includes individuals who report mild pain in the pain group (N=3,348 each cases and controls). Models include $age_{t=0}$, sex, birth year, education, wealth, chronic conditions, physical activity, alcohol consumption, smoking status, and wave 10 indicator.

Figure A1. Q-Q plots for model residuals compared with normal distribution.

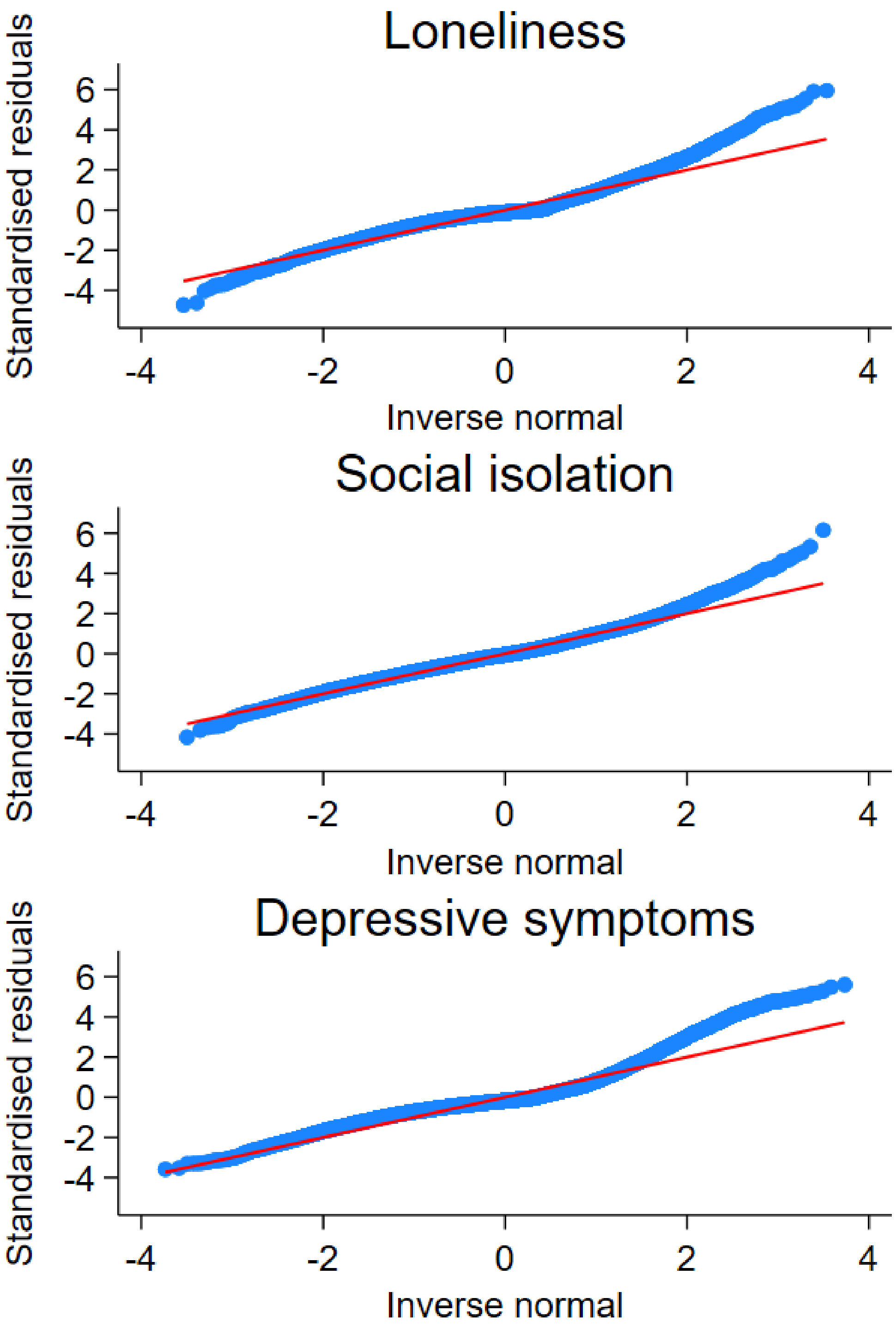

Figure A2. Loneliness score trajectory fitted using local polynomial regression.

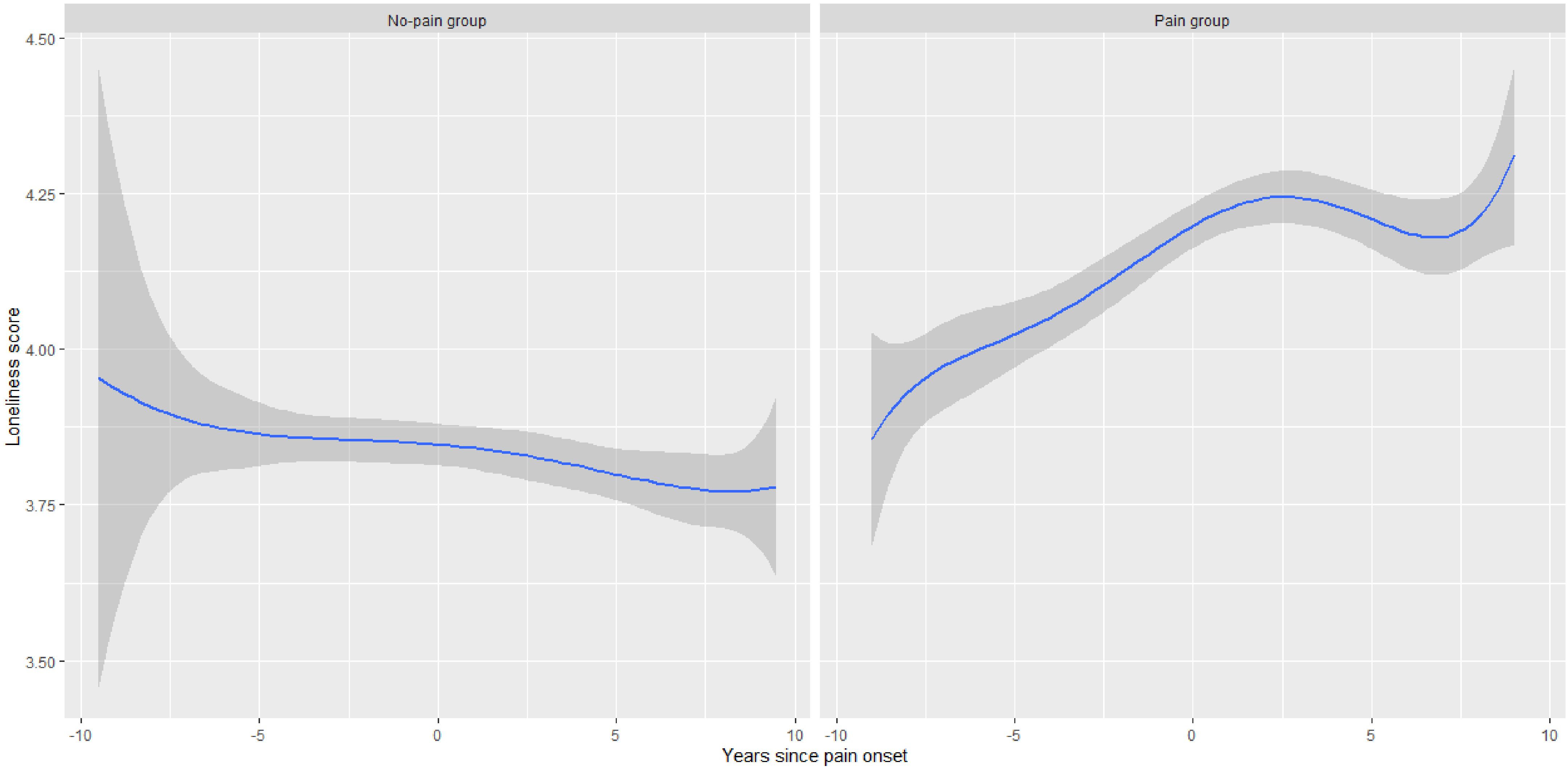

Figure A3. Social isolation score trajectory fitted using local polynomial regression.

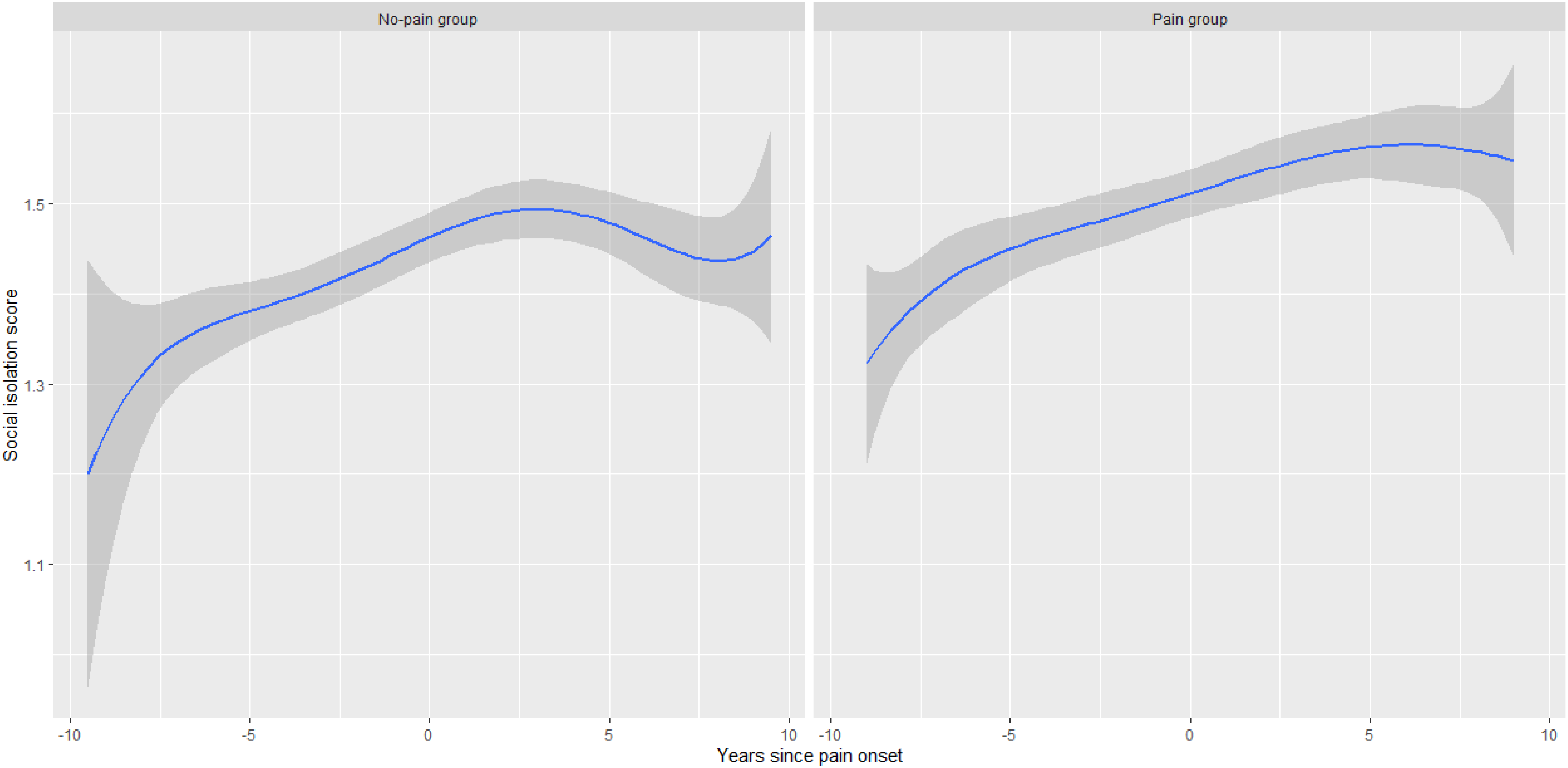

Figure A4. Depressive symptom score trajectory fitted using local polynomial regression.

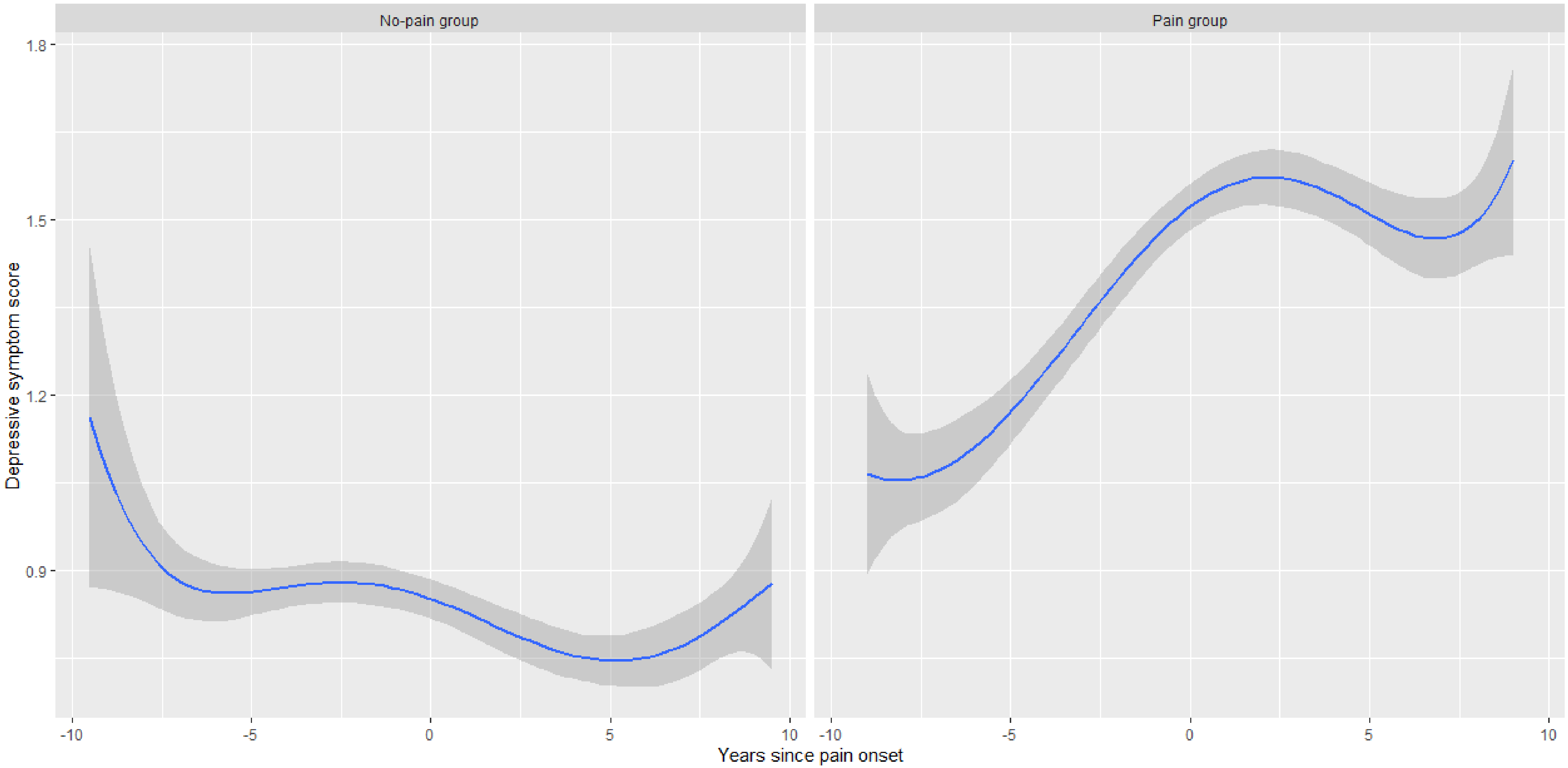

Figure A5. Flowchart of sample selection.

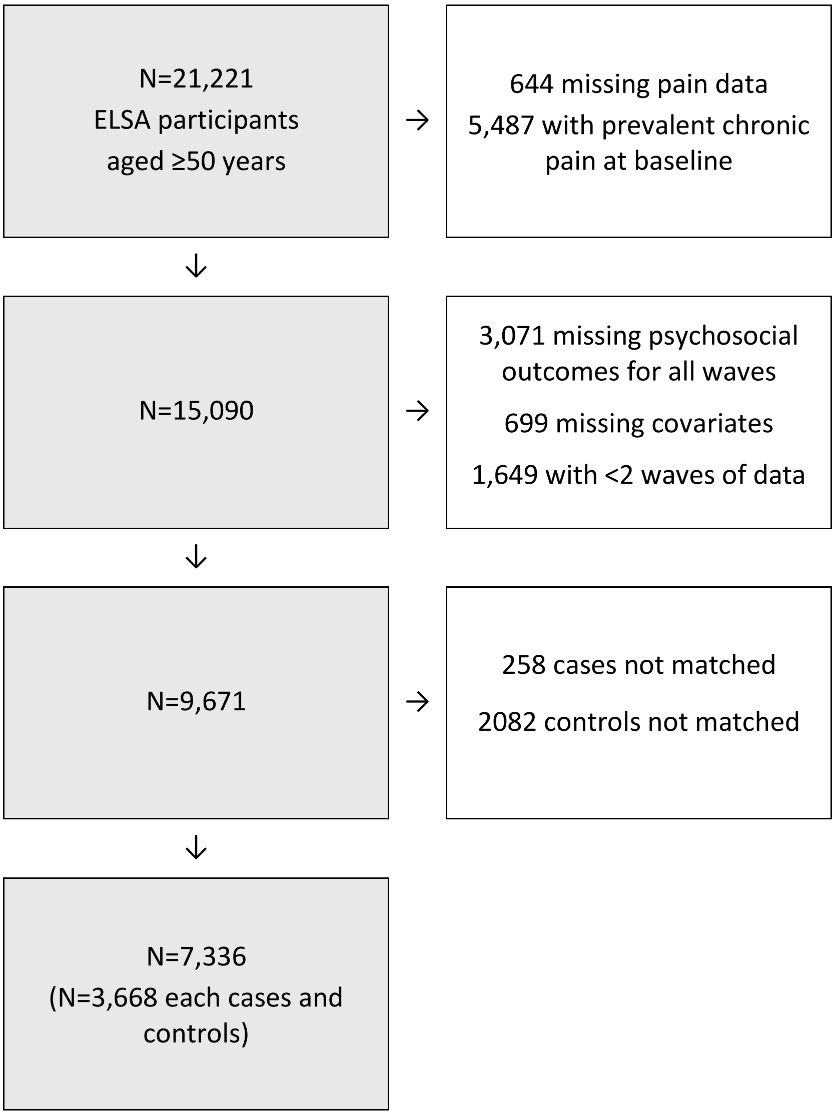

Figure A6. Distribution of psychosocial scores at baseline.

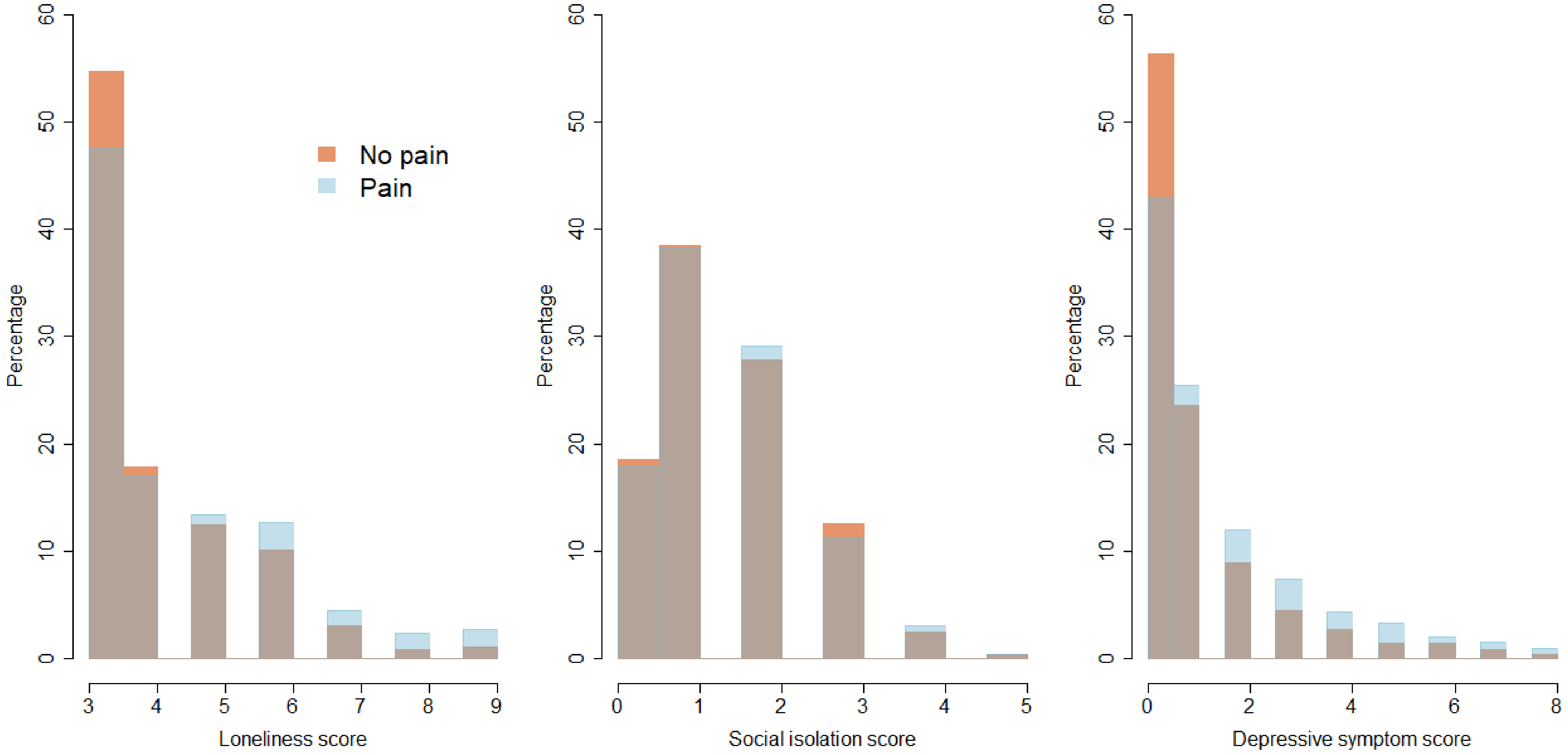

Figure A7. Trajectories of loneliness, social isolation, and depressive symptom scores before and after onset of pain (longer-term chronic pain only).

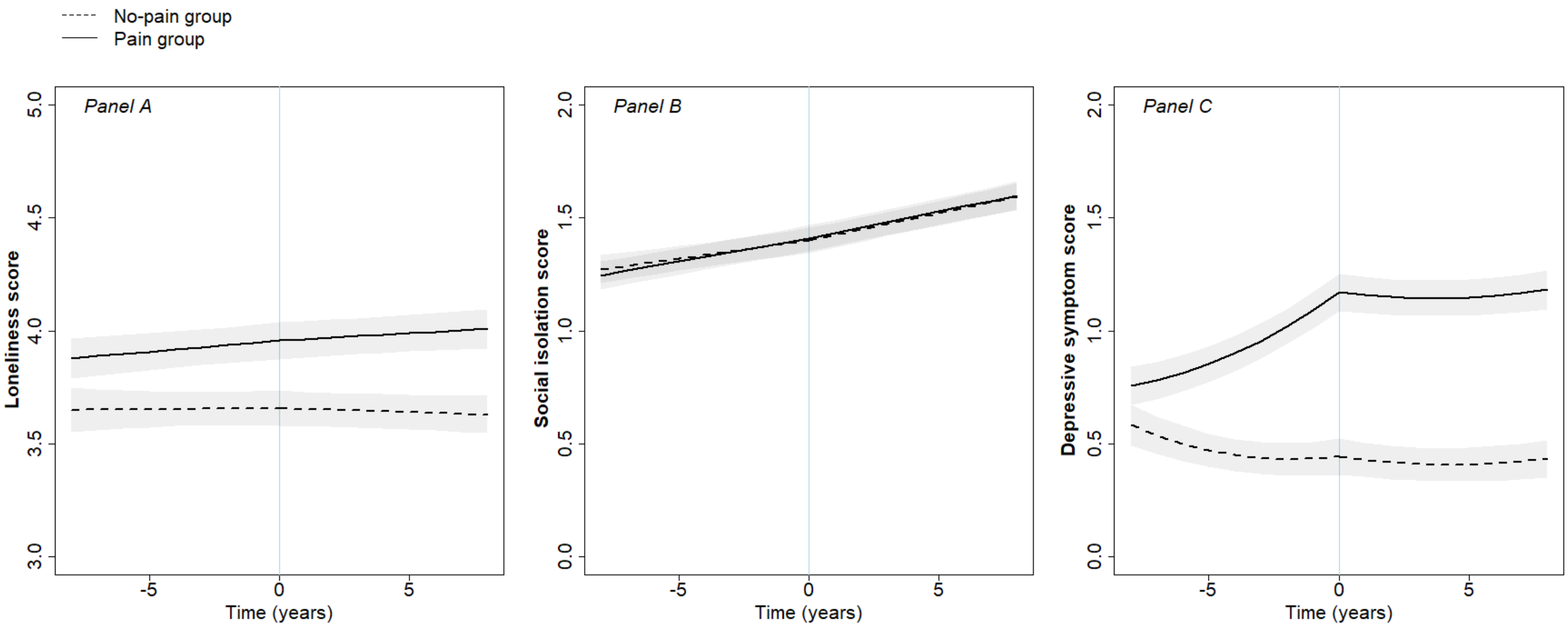


Panel A shows loneliness score (range: 3-9), panel B shows social isolation score (range: 0-5), and panel C shows depressive symptom score (range: 0-8). Models are adjusted for $age_{t=0}$, sex, birth year, education, wealth, chronic conditions, physical activity, alcohol consumption, and smoking status. Plotted for reference values of covariates ($age_{t=0}$=65, male, born 1940-49, high school diploma, mean wealth, no chronic conditions, weekly moderate-vigorous physical activity, consumes alcohol, non-smoker).

Figure A8. Trajectories of loneliness, social isolation, and depressive symptom scores before and after onset of pain (including mild pain).

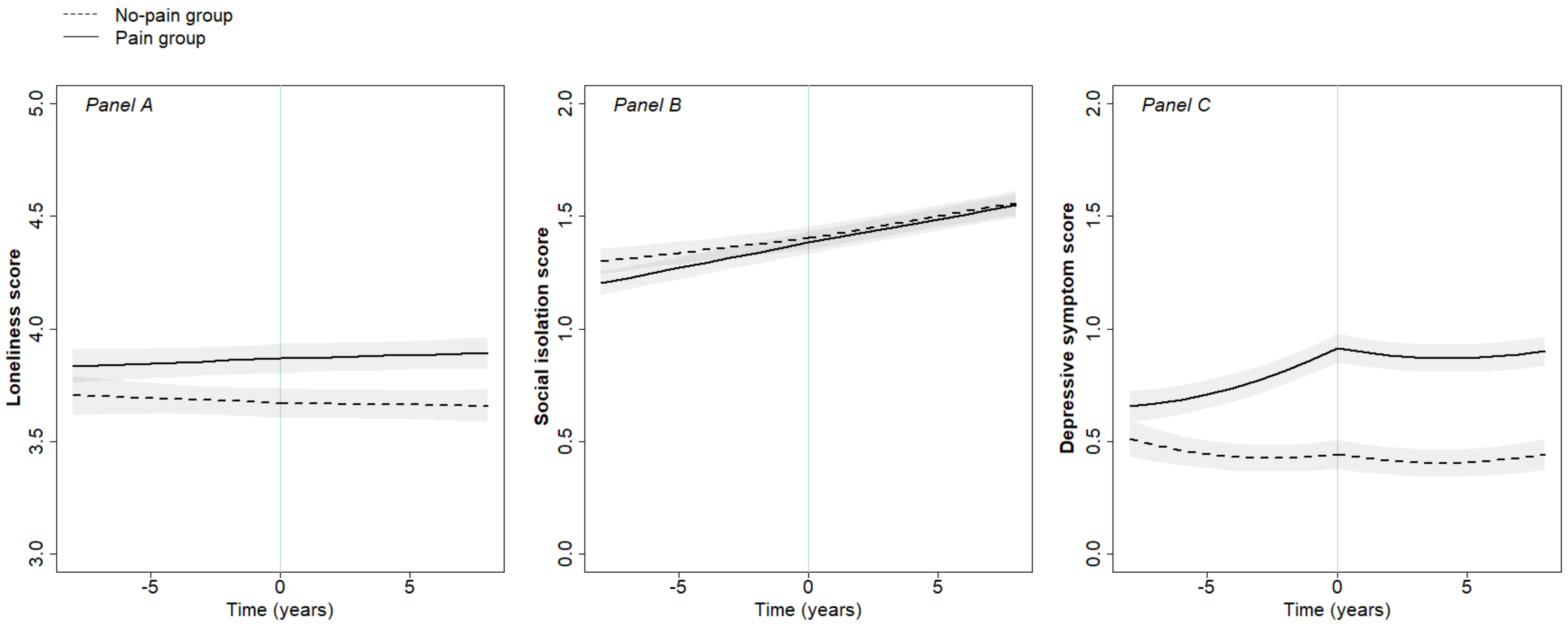


Panel A shows loneliness score (range: 3-9), panel B shows social isolation score (range: 0-5), and panel C shows depressive symptom score (range: 0-8). Models are adjusted for $age_{t=0}$, sex, birth year, education, wealth, chronic conditions, physical activity, alcohol consumption, and smoking status. Plotted for reference values of covariates ($age_{t=0}$=65, male, born 1940-49, high school diploma, mean wealth, no chronic conditions, weekly moderate-vigorous physical activity, consumes alcohol, non-smoker).

References

1. Hedeker D, du Toit SHC, Demirtas H, Gibbons RD. A note on marginalization of regression parameters from mixed models of binary outcomes. *Biometrics* 2018; **74**(1): 354-61.
